# Melting Down Protein Stability: PAPS Synthase 2 in Patients and in a Cellular Environment

**DOI:** 10.3389/fmolb.2019.00031

**Published:** 2019-05-03

**Authors:** Oliver Brylski, Simon Ebbinghaus, Jonathan W. Mueller

**Affiliations:** ^1^Institute of Physical and Theoretical Chemistry, Technische Universität Braunschweig, Braunschweig, Germany; ^2^Institute of Metabolism and Systems Research, University of Birmingham, Birmingham, United Kingdom; ^3^Centre for Endocrinology, Diabetes and Metabolism, Birmingham Health Partners, Birmingham, United Kingdom

**Keywords:** sulfation pathways, PAPS synthase, quinary interaction, excluded volume effect, ligand stabilization, enzyme storage complex

## Abstract

Within the crowded and complex environment of the cell, a protein experiences stabilizing excluded-volume effects and destabilizing quinary interactions with other proteins. Which of these prevail, needs to be determined on a case-by-case basis. PAPS synthases are dimeric and bifunctional enzymes, providing activated sulfate in the form of 3′-phosphoadenosine-5′-phosphosulfate (PAPS) for sulfation reactions. The human PAPS synthases PAPSS1 and PAPSS2 differ significantly in their protein stability as PAPSS2 is a naturally fragile protein. PAPS synthases bind a series of nucleotide ligands and some of them markedly stabilize these proteins. PAPS synthases are of biomedical relevance as destabilizing point mutations give rise to several pathologies. Genetic defects in PAPSS2 have been linked to bone and cartilage malformations as well as a steroid sulfation defect. All this makes PAPS synthases ideal to study protein unfolding, ligand binding, and the stabilizing and destabilizing factors in their cellular environment. This review provides an overview on current concepts of protein folding and stability and links this with our current understanding of the different disease mechanisms of PAPSS2-related pathologies with perspectives for future research and application.

## Introduction

Sulfation pathways are centered around enzymatic conversion of sulfate to the activated sulfate donor 3′-phosphoadenosine-5′-phosphosulfate (PAPS) and the transfer of the sulfuryl moiety to biological acceptor molecules (Foster and Mueller, 2018). The enzymes in charge are PAPS synthases (Mueller and Shafqat, 2013) and PAPS-dependent sulfotransferases (Coughtrie, 2016; Hirschmann et al., 2017), respectively. Sulfation pathways also include the removal of sulfate by sulfatases (Mueller et al., 2015) and the deactivation of the sulfation-byproduct 3′-phospho-adenosine-5′-phosphate (PAP) by dedicated PAP phosphatases (Chan et al., 2016). Sulfation impacts on many different acceptor molecules, such as carbohydrates, proteins, lipids, xenobiotics, and steroids as well as other hormones (Mueller et al., 2015). Several proteins from this pathway have been studied with regard to their stability; some of them giving rise to clinically observed pathologies (Oostdijk et al., 2015).

An experimental categorization into thermostable and thermolabile sulfotransferases (Reiter and Weinshilboum, 1982) was an early apprehension of the multiplicity of sulfation pathways we know of today. Unfolding was then monitored as loss-of-activity measurements upon thermal increase. Midpoints of unfolding of sulfation activity towards dopamine were 39.5°C, but 44°C for a phenol-targeting sulfotransferase (Reiter and Weinshilboum, 1982). Hence, thermostability was understood as being folded and active at about physiological temperature. This system was in use until more and more sulfotransferase genes were cloned (Dubin et al., 2001). Later, sulfotransferase proteins were purported to form dimers via an unusually small protein interface (Petrotchenko et al., 2001; Weitzner et al., 2009). Dimerization is thought to increase structural stability of human sulfotransferase SULT1A1, both with regards to thermal inactivation and chemically induced unfolding (Lu et al., 2009).

Only in 2012, the first biophysical study of sulfate-activating PAPS synthase enzymes (van den Boom et al., 2012) identified one of them, human PAPSS2, as a fragile protein specifically stabilized by ligand binding (van den Boom et al., 2012; Mueller and Shafqat, 2013). PAPS synthases are bifunctional enzymes comprising a C-terminal ATP sulfurylase and an N-terminal APS kinase domain (Mueller and Shafqat, 2013). Their physiological substrates and products are sulfate, ATP, ADP, pyrophosphate, PAPS, and the reaction intermediate adenosine-5′-phosphosulfate (APS) (Strott, 2002). Out of these, ADP and PAPS had moderately stabilizing effects, but APS shifted unfolding transitions by more than 16°C (van den Boom et al., 2012). The relevance of this finding for intracellular sulfation pathways is not yet clear as concentrations of the intermediate APS are expected to change considerably within the cell (Lansdon et al., 2004).

Various point mutations have been described for the *PAPSS2* gene that lead to bone and cartilage malformations (Oostdijk et al., 2015) as well as a steroid sulfation defect (Noordam et al., 2009; Oostdijk et al., 2015). A subset of these mutations seems to destabilize the PAPSS2 protein severely, inducing its intracellular aggregation and triggering its ubiquitination and degradation via the proteasome (Oostdijk et al., 2015). Interestingly, PAPSS2 is involved in transient protein interactions with other sulfation pathway proteins (Mueller et al., 2018) and these interactions may stabilize or activate the PAPSS2 protein. This review will look at sulfation pathways, central to healthy human physiology from a protein-stability/protein-folding perspective.

## PAPS Synthase Proteins Display Substrate-Specific Folding Properties

Understanding structure, function, and stability of proteins as the cellular workforce to generate vital biomolecules has been of great interest ever since (Bryngelson et al., 1995). Protein folding becomes especially important due to the many examples of malfunctioning proteins causally linked to severe diseases, such as Huntington's (McColgan and Tabrizi, 2018) and Parkinson's disease (Poewe et al., 2017). Many proteins are functional on their own, but intermolecular interactions such as dimer or multimer formation are common features of proteins (Marsh and Teichmann, 2015). These quaternary structures result from highly specific interactions encouraged by complementary surface properties of the proteins involved. They may represent the functional form of many proteins (Dobson et al., 2004), regulate activity (Grum et al., 2010) or be included in signal pathways (Heldin, 1995) and trafficking between compartments (Knauer et al., 2005; Schröder et al., 2012; Eggert et al., 2018). Compared to smooth interactions such as multimer formation, intermolecular protein interactions may also have a fuzzy nature. Interactions resulting in these fuzzy complexes usually involve intrinsically disordered regions that interact with each other to form for example signalosomes or phase-separating ribonucleoprotein granules (Wu and Fuxreiter, 2016; Alberti et al., 2019).

Within the complex environment of the living cell, transient interactions with other biomolecules may occur and these have been named quinary interactions (McConkey, 1982) as a continuation of primary, secondary, tertiary and quaternary structure (Cohen and Pielak, 2017). However, commonly biomolecules are probed in dilute buffer solutions or in crystals by techniques such as NMR spectroscopy or crystallography. Thus, protein functions relying on transient protein interactions mostly remain unnoticed or are very hard to study (Matena et al., 2013).

**Figure 1:**
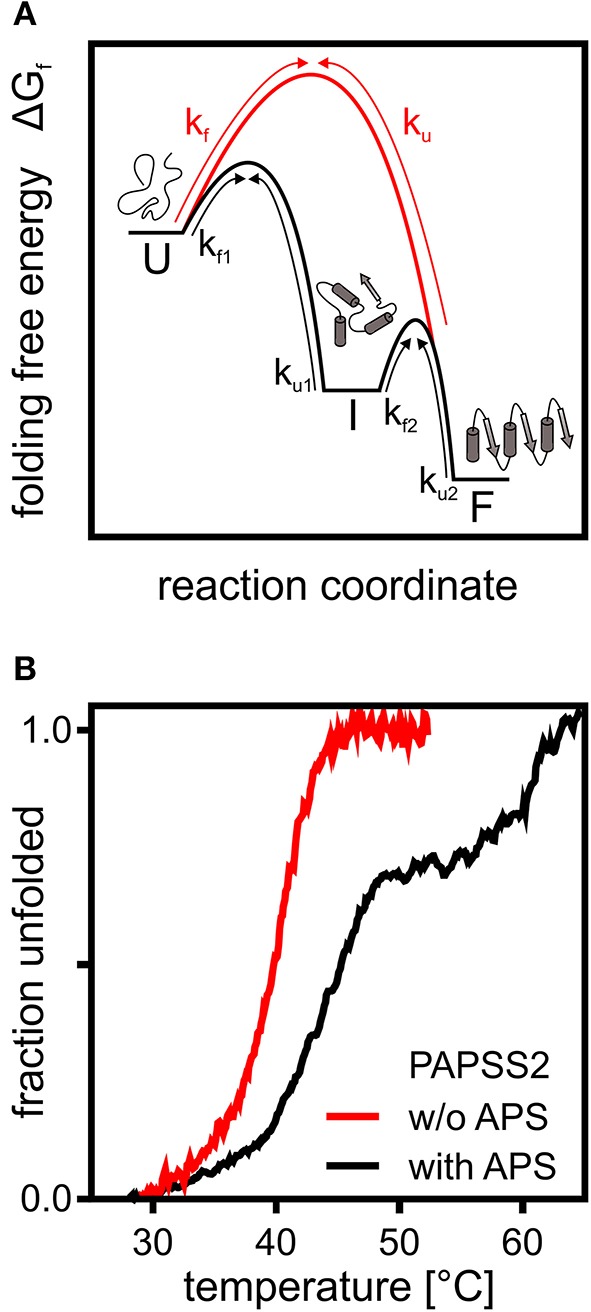
A theoretical and an experimental perspective on protein folding. **(A)** Schematic representation of the folding free energy landscape for a multi-state (black) and two-state folder (red). Kinetic constants for folding and unfolding (k_f_ and k_u_) are depicted with arrows highlighting the transitions between unfolded (U), intermediate (I), and folded (F) states. **(B)** Thermal CD unfolding data of PAPS synthase 2 adapted from van den Boom et al. (2012). Experiments without (red) and with (black) the APS nucleotide at a 100 fold excess of APS. Transitions observed fit the expected data for a two-state folder with one transition and a three-state folder with two distinguishable transitions. Multi-state behavior is only observed upon APS binding.

The different structural and interaction levels of proteins create a multidimensional rugged energy landscape with several small energetic minima representing different possible conformations of the polypeptide chain (Bryngelson et al., 1995). Even though the energy landscape in theory allows many marginally stable conformations, protein folding and unfolding of small single-domain proteins can often be described by a two-state model (Figure 1A). The unfolded protein needs to cross a transition state comprised of an ensemble of partially folded structures in order to fold downhill into its native conformation, by forming intramolecular interactions in a cooperative manner (Bryngelson et al., 1995; Oliveberg and Wolynes, 2005). This one-dimensional energy landscape model allows to precisely determine rates of protein folding as a folding “speed limit” (Kubelka et al., 2004). The influence of intermolecular and intramolecular interactions as well as pH or viscosity on folding and the ruggedness of the landscape are still investigated extensively (Chung et al., 2015; Chung and Eaton, 2018). However, the simple model needs adjustments when describing folding of large multi-domain proteins. Multi-domain proteins may form stable and biologically relevant intermediates representing distinct energy minima on the protein folding energy landscape. This applies to proteins with independently folding domains (Ferreiro et al., 2005) or those requiring specific interactions with substrates or cofactors (Klein and Schwarz, 2012; van den Boom et al., 2012). The actual positioning on the reaction coordinate and their stability compared to the unfolded and folded state may differ according to the number of contacts already formed in this structure. PAPS synthases with their two independently folding domains are a good example for multi-state folder.

The 140 kDa dimeric PAPS synthase proteins unfold irreversibly thermally and chemically, when studied as recombinant proteins *in vitro* (van den Boom et al., 2012). Their reaction intermediate, the nucleotide adenosine-5′-phosphosulfate, has two effects on the unfolding thermographs (Figure 1B). At low APS concentrations, equal to or slightly higher than the protein, unfolding transitions shift remarkably by about 5°C. At higher APS concentrations, even the form of the unfolding transition changed and a clear unfolding intermediate is seen (van den Boom et al., 2012). The high-affinity effect can be explained by forming a stable dead-end enzyme-ADP-APS complex in the APS kinase domain (Mueller and Shafqat, 2013), further discussed below. The ADP for this complex most likely is carried over through the purification process as it is tightly enzyme-bound (Mueller and Shafqat, 2013). The low-affinity effect is most likely due to stabilization of the ATP sulfurylase domain (van den Boom et al., 2012). In summary, this shows that PAPS synthase proteins display complex and substrate-specific folding properties that could play a important role in the regulation of sulfation pathways. It will be crucial to study PAPS synthase stability in a cellular environment with tightly regulated substrate concentrations and the crowded environment described below.

## Push and Pull—Crowded Cells vs. Buffer Solutions

In comparison to dilute solution, the cellular environment is crowded with up to 300 g/L of biopolymers (Zimmerman and Trach, 1991; Ellis and Minton, 2003). Biomolecules in such environments are influenced by a many of specific and non-specific interactions. Metaphorically speaking, comparing a biomolecule in dilute solution to the situation in the cell is like comparing an elegant ballet dancer in an empty dancing hall with a person in an overcrowded night-club. Random interactions and collisions are rare in the first case, but unavoidable in the second. Scientifically speaking, the consequences of the random interactions and the collisions in the cellular “night-club” are referred to as excluded-volume effects (Minton, 1983) and quinary interactions (McConkey, 1982), respectively.

Within the concept of excluded-volume, macromolecules primarily repel each other. High macromolecule concentrations increase the occupied volume and decrease the volume accessible for other macromolecules, hence, reducing the configurational entropy of less compact conformations. With regards to proteins, this favors compact protein conformations that normally are the functional protein folds (Minton and Wilf, 1981). Indeed, *in vitro* experiments show that an artificially crowded environment using large polymers such as PEG or Ficoll-70 stabilizes native conformations (Alfano et al., 2017) and favors protein association (Batra et al., 2009) and catalytic activity (Dhar et al., 2010; Paudel et al., 2018). However, a physicochemical force driving proteins into their most compact conformation will unavoidably also affect aggregation-prone proteins and increase aggregation rates in a crowded environment (Hatters et al., 2002; Gao et al., 2015).

The concept of quinary interactions describes the organization of large biomolecules via weak and transient interactions (McConkey, 1982). In contrast to repulsive interactions, transient interactions depend on the chemical composition of the biomolecule and the crowding agent (Minton, 2013). Since crowding agents, used to mimic the cellular environment, differ in their chemical properties, their effects on a target protein studied *in vitro* may hence differ as well (Senske et al., 2014; Feig et al., 2017; Majumdar et al., 2018). While some crowding agents such as PEG directly interact with a protein, others such as dextran rather affect the water network and the solvation of the protein (Senske et al., 2014). This mechanism is comparable to the thermodynamic stabilization of proteins by osmolytes (Yancey et al., 1982; Senske et al., 2014, 2016). Recently, the effects of quinary interactions on protein stability have been correlated with protein surface properties – the increase in surface charge is a key factor for the formation of quinary interactions (Gnutt et al., 2019). These well-studied concepts show that in addition to the intrinsic stability of the protein fold, weak and repulsive interactions in a cellular environment need to be considered as well for a complete view on protein folding and stability. Detailed effects of the cellular environment and how intracellular crowding is mimicked *in vitro* were discussed here (Politou and Temussi, 2015; Gnutt and Ebbinghaus, 2016).

Understanding how a protein folds and how it is stabilized, when surrounded by hundreds of other macromolecules with a myriad of unspecific interactions is a complex task. Several approaches have been applied to disentangle the physicochemical effects exerted on biomolecules inside the cell, ranging from fluorescent crowding sensors (Boersma et al., 2015; Gnutt et al., 2015) to case-studies in cell lysates (Martin and Hartl, 1997) or whole cell simulations (Feig et al., 2017). Within living cells, similar studies have been performed using an RNA hairpin (Gao et al., 2016), the glycolytic PGK enzyme (Dhar et al., 2011; Wirth et al., 2013), superoxide dismutase 1 (Gnutt et al., 2019), cell volume changes (Wang et al., 2018), and intracellular osmolytes (Sukenik et al., 2018). Very recently, the repertoire of in-cell protein analysis has been expanded to live zebrafish (Feng et al., 2019). It appears that the net-effect on thermal stability of a specific protein or sensor inside cells cannot be generalized and highly depends on the surface exposed. This complexity makes it inevitable to use in-cell techniques to study proteins within their native environment complemented with *in vitro* experiments.

An orthogonal method to study in-cell protein stability represents mass-spectrometry of isotopically labeled cells. A pioneering study in this regard reported thermal profiling of the cellular proteome (Savitski et al., 2014). From this dataset, we derived midpoints of unfolding T_50_ of 54.6 and 46.2°C for PAPSS1 and PAPSS2, respectively, and compared these to the T_50_ values of 45.8 and 39.8°C for PAPSS1 and PAPSS2, measured before (van den Boom et al., 2012), revealing a difference in T_50_ of 8.3 and 5.8°C for the two PAPS synthases. Even though these numbers are from two studies using different methods, the observed differences in T_50_ suggest that PAPS synthase protein stability within cells may differ compared to previous *in vitro* studies (van den Boom et al., 2012). Whether this is related to intrinsic protein stability or protein interactions is a question which remains to be answered.

## Holding on to Something—How Ligands, Cofactors and Substrates Affect Protein Stability

Functional proteins have not evolved to preserve a certain protein fold, but rather to fulfill a certain biological function. Enzymes have specific binding sites for strong and specific interactions with their small-molecule substrates and products (Martínez Cuesta et al., 2015). This specific binding relies on many cooperatively acting interactions and may stabilize the protein, making it more rigid. Indeed, many experiments studying thermal or mechanical stability show strong stabilization of proteins by binding their substrates or cofactors. This includes, among others, adenylate kinases binding substrate and inhibitory nucleotides (Mazal et al., 2018), PAPS synthases binding their substrates or products ADP, APS, and PAPS (van den Boom et al., 2012) as well as cohesins binding calcium (Verdorfer and Gaub, 2018). But how do these newly formed interactions contribute to the actual stability of the protein?

The Gibbs energy ΔG is genuinely used to compare the energetic differences between two states and in protein-ligand-binding this refers to the bound and the unbound state of the ligand. The Gibbs energy can further be split into contributions of the enthalpy ΔH describing the total energy of the system observed and the temperature-dependent entropy ΔS describing the degrees of freedom or conformational states of similar energy. The thermodynamic descriptions of protein-ligand binding focus either on the ligand, especially in drug design (Claveria-Gimeno et al., 2017), or how the energy landscape of protein folding and stability is affected (Kabir et al., 2016; Hingorani et al., 2017). One factor regarding ligand binding is the process of breaking existing water networks in the unbound state in order for the ligand to bind (Fox et al., 2017; Verteramo et al., 2019). Breakage and reorganization of a strong water network displays an energetic cost which ultimately reduces the affinity of drugs to their targets (Fox et al., 2017). Thermodynamic analysis focusing mainly on the binding effects on the protein itself include changes in protein structure (Reyes et al., 2017) or stability (Kabir et al., 2016). In some cases, binding of ligands may actually stabilize non-native or intermediate conformations and therefore not always leads to a stabilization of the protein target (Kabir et al., 2016). However, stabilizing non-native interactions may be desirable in drug design to reduce formation of, for example, toxic aggregates (Vöpel et al., 2017; Perni et al., 2018).

**Figure 2:**
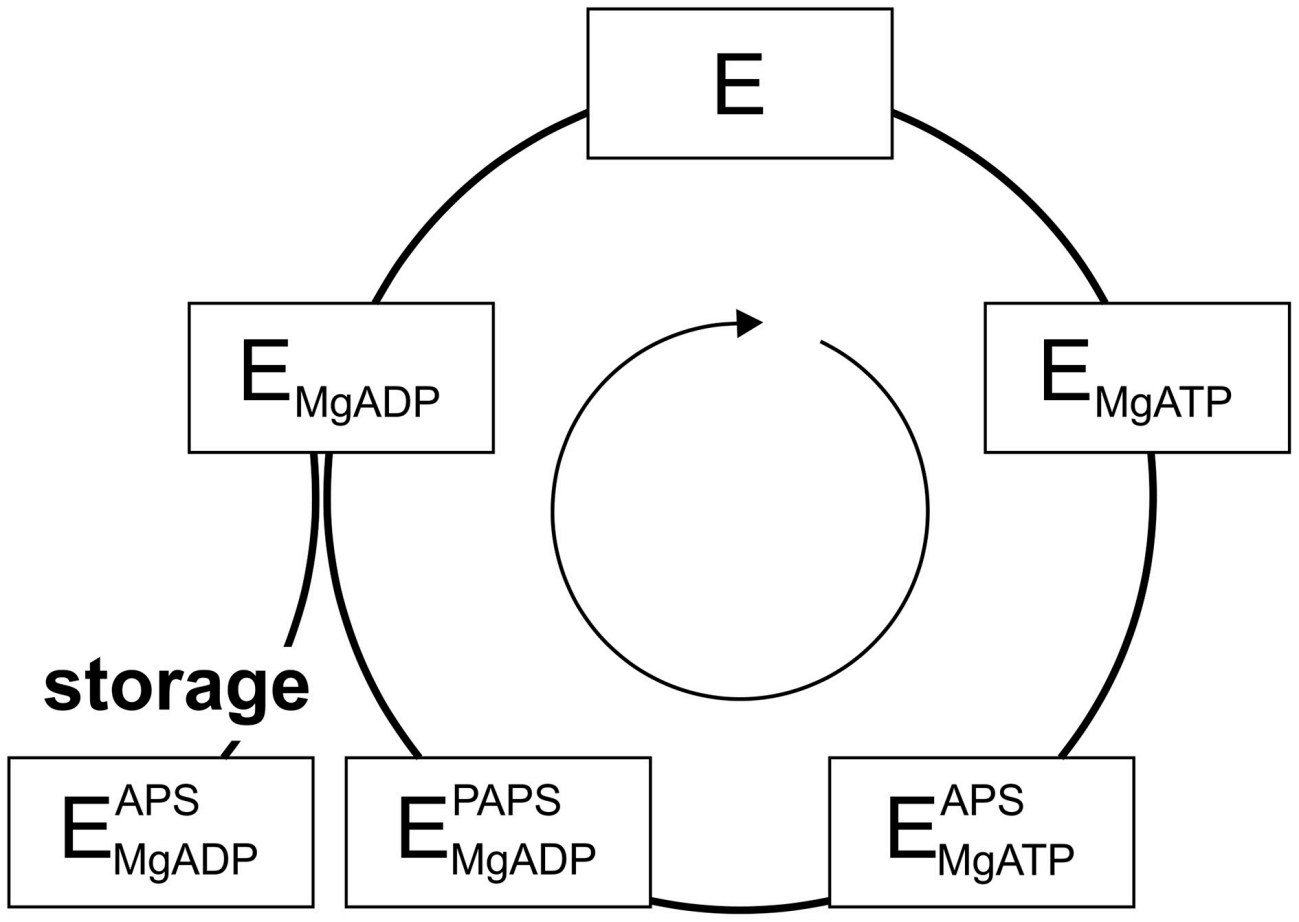
The catalytic cycle of APS kinase. Schematic representation including substrate binding and product release steps. An inhibitory or stable storage complex is populated after PAPS release by binding of APS (lower left).

PAPS synthases show consecutive binding and release of sulfate, ATP, ADP, APS, and PAPS (Sekulic et al., 2007; Figure 2). As all nucleotides involved are generated from ATP, it becomes apparent that ATP availability fuels the cycle. Looking into crystal structures and experimental data, ADP and APS appear to be tightly bound to the protein (Harjes et al., 2005; Mueller and Shafqat, 2013) and the structure of the protein is known to be stabilized by these nucleotides (van den Boom et al., 2012). The inhibitory or “dead-end” complex forms after the release of the product PAPS, by re-binding of an APS nucleotide to the ADP-kinase complex. This is a highly stabilized form of the APS kinase (Sekulic et al., 2007; Mueller and Shafqat, 2013). Considering thermal unfolding experiments in presence and absence of APS described above (van den Boom et al., 2012), one of the observed thermal unfolding transitions must be caused by the formation of this inhibitory complex with APS (Figure 1B). Due to the marked increase in stability by binding ADP and especially APS, the formed complex may not only be of inhibitory nature, but serves as a storage form in times of ATP depletion.

Even though ATP is often thought to be a ubiquitously available source of energy inside cells at up to two-digit millimolar concentration, the levels of ATP are regulated by different factors and may fluctuate significantly during the lifetime of a cell (Beis and Newsholme, 1975; Ataullakhanov and Vitvitsky, 2002; Bonora et al., 2012). However, in response to ATP depletion resulting from stresses, kinases such as 5′-AMP protein kinase become more active in order to rebalance the ATP/AMP-ratio by phosphorylating substrates, regulating downstream gene expression and ultimately the activity of ATP-generating systems (Hardie and Carling, 1997). DNA damage from irradiation and oxidative stress caused by endogenous or environmental factors are stresses that affect ATP levels temporarily (Budanov and Karin, 2008). However, hyperosmotic shock (Dmitrieva et al., 2011), resulting in an imperfect crowding adaptation (Gnutt et al., 2017), as well as cellular starvation (Maddocks et al., 2013) are known to cause DNA lesions as well. Further, as ATP has been suggested to act as a hydrotrope helping to solubilize proteins in the cellular environment, proteins may even become prone to aggregation, if ATP is depleted for a longer period of time (Patel et al., 2017). Considering changing ATP levels and PAPS synthase stabilization via ligand binding, we propose that the PAPSS2-ADP-APS complex within the APS kinase domain represents a stable storage form not only of the enzyme, but also for the APS nucleotide, of relevance in times in which ATP levels are depleted.

## Intracellular Recycling—Stability and Degradation of PAPS Synthases in Cells

Proteins within the cell are not only exposed to the physicochemical forces described above, but also to enzymatic post-translational modifications and some of these modifications influence protein stability again. In Alzheimer's disease, as an example, the phosphorylated tau protein forms neurofibrillary tangles and glycosylation defects have been observed in the amyloid precursor protein (APP) and other proteins (Schedin-Weiss et al., 2014). Ubiquitination is noteworthy here as it serves, among other functions, to target proteins for active degradation (Chaugule and Walden, 2016). A tetra-ubiquitin chain attached to a lysine side chain of a target protein is sufficient for recognition and degradation by the proteasome (Singh et al., 2016). The attachment of ubiquitin is catalyzed by hundreds of ubiquitin ligase-complexes (Chaugule and Walden, 2016). Interestingly, this attachment depends on physicochemical stability of the target protein as ubiquitination requires the target peptide to be unfolded or intrinsically disordered (Prakash et al., 2009); structured domains need to unfold partially before ubiquitination can occur (Hagai et al., 2011) and conversely ubiquitination destabilizes the protein fold (Hagai and Levy, 2010). Preliminary data for PAPS synthases indeed show enhanced ubiquitination for PAPSS2 than for PAPSS1 upon 6 h treatment with the proteasome inhibitor MG-132 (data not shown). Taken together, biophysical instability may translate to biological instability involving active degradation by the ubiquitin/proteasome machinery.

## One Protein, Different Mutants and Two Mechanisms—Understanding PAPS Synthase-Related Diseases and Applying This Knowledge to Drug Design

Several neurodegenerative diseases present with aggregates of certain proteins such as Aβ peptide in Alzheimer's disease (Masters et al., 2015), poly-Q repeats in Huntington's disease (McColgan and Tabrizi, 2018), and α-synuclein in Parkinson's disease (Poewe et al., 2017). Once disease progression was linked to the cytotoxicity of the observed protein aggregates, aggregation had been studied in detail and the actual toxic oligomeric species identified (Knowles et al., 2014). Most recently, small molecules have been developed to alter aggregation properties of the affected protein to treat the according disease (Vöpel et al., 2017; Perni et al., 2018; Limbocker et al., 2019).

**Figure 3:**
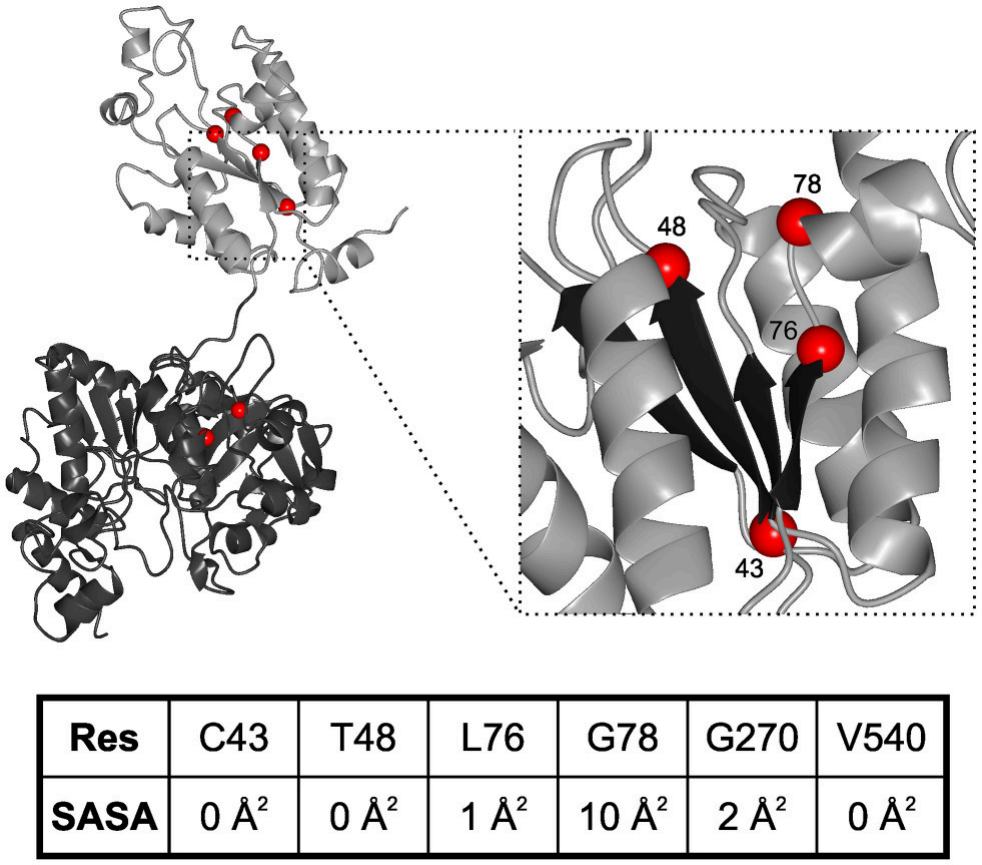
Structural mapping of PAPSS2 point mutations. Known disease-relevant mutations in PAPS synthase 2 isoform (red spheres) are shown in a PAPS synthase structure (PDB: 1XNJ). The N-terminal APS kinase is shown in gray, the C-terminal ATP-sulfurylase domain in black. **Zoom**, disease relevant mutations in the APS kinase central beta-sheet (black). Amino acid numbering reflects positions in PAPSS2. **Table**, solvent accessible surface area (SASA) of sidechains derived from DSSP values. Values for amino acids in the APS kinase domain have been derived from PDB structure 2AX4, values for the ATP sulfurylase are from 1XNJ.

While neurodegenerative diseases are well-studied, diseases related to sulfation pathways remained underexplored with the exception of heparin as an anti-coagulant (Tsai et al., 2017). Furthermore, genetic defects in the gene for PAPS synthase 2 have been described with phenotypes of various forms of bone and cartilage formation as well as androgen regulation and polycystic ovary syndrome (Oostdijk et al., 2015). These PAPSS2 missense mutations include G78R (Kurima et al., 1998), T48R (Noordam et al., 2009), C43Y, L76Q, V540D (Iida et al., 2013), and G270D (Oostdijk et al., 2015). They are mapped on a schematic representation of the PAPS synthase protein structure (Figure 3), which shows that the APS kinase mutants are in close spatial proximity.

Some of the mutations in PAPSS2 occurred homozygous such as V540D (Iida et al., 2013; Oostdijk et al., 2015). However, two studies describe clinical cases of compound heterozygous mutations, where a missense mutation is observed in the genetic background of a non-sense mutation (Noordam et al., 2009; Oostdijk et al., 2015). Messenger RNAs of non-sense mutations with their premature stop codon are substrates for non-sense-mediated mRNA decay (Lloyd, 2018) and hence they are regarded as *null* alleles. For three of the missense mutations, G78R, T48R, and G270D, it is known that they are hypomorphic; they are alleles with residual activity. For the G78R variant, very little residual APS kinase activity was reported, but the ATP sulfurylase activity remained similar to wild type (Kurima et al., 1998), suggesting a folded and functioning ATP sulfurylase that may act as a solubility anchor and prevent immediate aggregation of the protein. For T48R and G270D, residual ability to support SULT2A1-dependent DHEA sulfation was reported, however, both mutated protein variants were also ubiquitinated and degraded (Oostdijk et al., 2015), suggesting severely destabilized protein folds.

These data suggest the existence of two different disease mechanisms for the same PAPS synthase proteins. Residue solvent accessibility extracted from DSSP values further illustrates that G78R is more solvent exposed compared to the buried amino acids (keeping in mind that glycine residues show small solvent accessible surfaces in general due to the lack of a side chain). For an understanding of PAPSS2-related pathologies, it will be essential to investigate whether biophysical observation can be correlated with disease progression.

Generally, once a protein's molecular disease mechanisms is classified as misfolding or aggregation, designing small molecule-drugs is often the best way to recover that protein from its disease states (Arosio et al., 2014; Vöpel et al., 2017; Chia et al., 2018). This may be applicable to any other disease that compromises the protein-folding homeostasis (Wood et al., 2018). However, if the catalytic function of the protein is affected, this approach appears not promising and cure is probably best to achieve by supplementing patients with the lacking compounds that are no longer synthesized by the affected protein. For defects in the biosynthetic pathway of a cofactor, such supplementation can involve a precursor of that cofactor (Edwards et al., 2015); for PAPS synthase defects it may turn out to be a small subset of sulfated biomolecules.

## Conclusion and Outlook

Activated sulfate in form of PAPS is essential for driving sulfation pathways inside cells. Even though a lack of PAPS synthase activity results in severe disease states, biophysical studies have only focused on measurements in buffered solutions so far. These findings implicate that PAPS synthases may be highly influenced by the cellular interior with regard to ligand binding and protein ubiquitination and degradation. At times, PAPS synthases form an inhibitory complex with ADP and APS bound. This complex may be reinterpreted as a storage form of the intrinsically instable PAPSS2 protein and its substrate APS. In-cell studies are required to determine the intracellular stability of PAPS synthases. These findings shall provide further insight into proposed molecular disease mechanisms and help to design suitable drugs or treatment strategies of PAPS synthase-related diseases.

## Author Contributions

SE and JWM defined the scope for this review. OB and JWM collected material and wrote the paper. All authors read and approved the final version of this review.

### Conflict of Interest Statement

The authors declare that the research was conducted in the absence of any commercial or financial relationships that could be construed as a potential conflict of interest.
